# Pore-Selective Fullerene Loading in a Porphyrin-Based Metal–Organic Framework Controls Photoinduced Charge-Transfer Dynamics

**DOI:** 10.1021/acs.jpclett.6c00574

**Published:** 2026-04-09

**Authors:** Alison Arissa, Thomas Rose, Noémi Leick, Pavel Kucheryavy, Junjie Ouyang, Huixin He, Stefan Grimme, Justin C. Johnson, Jenny V. Lockard

**Affiliations:** † Department of Chemistry, 67206 Rutgers University-Newark, Newark, New Jersey 07102, United States; ‡ Max-Planck-Institut für Kohlenforschung, D-45470 Mülheim an der Ruhr, Germany; § Mulliken Center for Theoretical Chemistry, Clausius-Institut für Physikalische und Theoretische Chemie, 9374 Rheinische Friedrich-Wilhelms Universität Bonn, Bonn 53115, Germany; ∥ 53405 National Laboratory of the Rockies, 15013 Denver West Parkway, Golden, Colorado 80401, United States

## Abstract

Building porous donor–acceptor networks based on host–guest interactions in metal organic frameworks (MOFs) provides unique opportunities for tuning charge separation in highly tailorable materials. Here we focus on installing electron-rich porphyrins and electron-deficient fullerene derivatives in the PCN-222 MOF using a solvent assisted ligand insertion (SALI) method. The fullerene is primarily bound in the large pore, where it is subject to distinct dielectric environments through dimethylformamide (DMF) and 1,4-dioxane solvent exchange. Following photoexcitation, sub-picosecond charge transfer involving initial exciplex population is observed, with different charge recombination pathways and lifetimes depending on solvent polarity through modulation of charge-transfer state energies. While the 1,4-dioxane environment yields charge recombination within 1 ns via local fullerene and porphyrin triplet state population, DMF results in charge recombination directly to the ground state on much longer time scales, including some lifetime components in the microsecond range. Fullerene loading influences these kinetics, and the potential for charge delocalization due to fullerene aggregation within the pores is evaluated by using molecular dynamics simulations.

## Graphical abstract

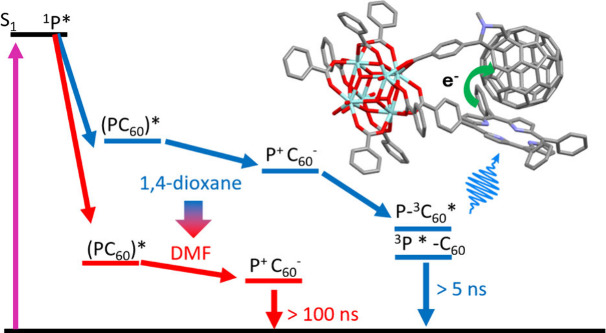

Metal–organic frameworks (MOFs) have tremendous potential as tunable optoelectronic and photocatalytic materials with high chemical versatility due to their inherent porosity and nearly infinite combinations of building blocks. Intensive research into MOF topologies has uncovered structural classes that possess excellent stability, particularly those composed of high valent metal-oxo nodes. A desirable strategy that builds on these known stable MOFs but modulates function involves utilizing their innate permanent porosity and pore-accessible reactive sites through postsynthetic modification (PSM) with nonstructural elements. One promising approach is solvent-assisted ligand incorporation (SALI), which adds certain chemical species to metal-cluster node sites. Most commonly, SALI has been demonstrated through binding to terminal hydroxides at the Zr-oxo cluster of the UiO-66, NU-1000, and PCN-22x MOF classes.^1,2^ SALI has been shown to add a variety of species in the pores of these frameworks, including metal complexes and photo- and redox active chromophores.^2^

One particular property of interest for advancing applications is the generation of long-lived and potentially mobile charges. Approaching this challenge through simple intercalation of a charge acceptor into a MOF has shown progress but is typically nondeterministic.^3–5^ We recently demonstrated the postsynthetic incorporation of fullerene in the well-established porphyrin-based MOF, PCN-222, and its participation as an electron acceptor upon photoexcitation of the porphyrin linkers which serve as electron donors.^6^ While relatively long-lived charge separation was observed, confinement of the fullerene guests within the small pores of the MOF via van der Waals forces displaced the pore-occupied solvent in the process and effectively minimized tunability of the dielectric environment of the donor–acceptor pair toward the generation of long-lived and/or mobile charges. Our goal in this work is to use SALI to incorporate fullerene derivative acceptors into PCN-222 such that they remain solvent accessible upon interaction with the porphyrin linkers. Prior work in this area suggests SALI leads to fullerene binding in this and related MOF structures and allows for some control over photophysics/catalysis.^7,8^ However, details of excited-state dynamics and structure–function relationships are lacking. Here, covalent binding through SALI places the fullerene moiety in the large pore, where dielectric effects from changing solvent lead to dramatically different outcomes after photoexcitation, including the formation of long-lived charge-transfer (CT) states in a solvent of high polarity. Using a variety of structural and spectroscopic probes, we quantify the amount and distribution of C_60_ loading and confirm covalent binding via SALI through spectral perturbations. Likely donor–acceptor geometry is verified via density functional theory (DFT) optimizations. We probe the photodynamics of this donor–acceptor framework through optical transient absorption spectroscopy and conclude, with the support of molecular dynamics simulations, that control of the various arrangements of components in the MOF (including fullerene/porphyrin, fullerene/fullerene, and the solvent environment) is crucial toward engendering long-lived and potentially mobile charges.

Following the successful synthesis of PCN-222 nanoparticles using established literature precedent,^6,9^ the carboxylic acid-functionalized fullerene derivative commonly known as C_60_-SAM is incorporated into the framework using the SALI method.^1^ After thorough washing to remove unbound C_60_-SAM molecules, powder X-ray diffraction (PXRD) characterization of the C_60_-SAM⊂PCN-222 framework (Figure S1) confirmed the crystallinity and phase retention of the PCN-222 structure upon postsynthetic modification. In the SALI process, C_60_-SAM replaces terminal hydroxyl and aqua groups on the Zr-oxo cluster, forming a bidentate carboxylate-appended structure. The exclusive orientation of the Zr-oxo-bound hydroxyl groups toward the large hexagonal channels of PCN-222 suggests that these pores serve as the sole location for accommodating the covalently attached C_60_-SAM guests.

The total amount of C_60_-SAM loaded into the porous structure after extensive washing was quantified using a previously reported weighing method that relies on the insolubility of fullerene derivatives under MOF digestion solvent conditions.^7,8^ Details of the procedure are provided in the Supporting Information. Using this method, the loaded amount of C_60_-SAM was determined to be 1.33 molecules per Zr-oxo node. Assuming exclusive coordination within the hexagonal channels of the MOF, this loading amount equates to an average ratio of 0.67 C_60_-SAM guest per porphyrin linker site. This may be an upper limit as some minor portion of the fullerene species may be located in the small triangular pore of the MOF. As another check on the SALI-introduced fullerene loading level, we turned to quantitative NMR characterization,^10^ which involves measurement of C_60_-SAM⊂PCN-222 after digestion with an internal standard. Details of the procedure are provided in the Supporting Information along with the NMR results and analysis that revealed a 0.55:1 C_60_-SAM to porphyrin ratio in the fullerene-loaded framework.

While the mass difference and qNMR methods yield an estimated range of the total loading of the fullerene derivative, nitrogen (N_2_) physisorption isotherms (Figure S3) provide insight into the dominant pore location of the introduced guests. The BET N_2_ isotherms reveal a decrease of PCN-222 surface area from 863.5 ± 5.1 to 650.9 ± 3.3 m^2^/g after C_60_-SAM intercalation. The pore size distribution (PSD) of the MOF before and after the SALI procedure and the cumulative pore volume analyses obtained from the physisorption isotherms using a DFT model are shown in Figure 1a. The higher cumulative pore volume and surface area of PCN-222 compared to those of the fullerene-loaded framework, C_60_-SAM⊂PCN-222, logically follow guest intercalation. PSD of the PCN-222 MOF shows two pore channels with ∼12 Å (triangular pore) and ∼34 Å (hexagonal pore) diameter, consistent with previously reported pore volume distributions for this framework.^11^ While the small pore volume of these frameworks decreased by only 14% for C_60_-SAM⊂PCN-222, the volume of the large hexagonal pores decreased by a far more significant 46%. By comparison, loading these MOFs with C_60_ (or PCBM), i.e., fullerene guests not susceptible to Zr-node attachment via SALI, as previously reported,^6^ led to more substantial reduction of the small-pore versus the large-pore volumes. These results confirm the preferentially large pore location of C_60_-SAM following the SALI method.

**Figure 1:**
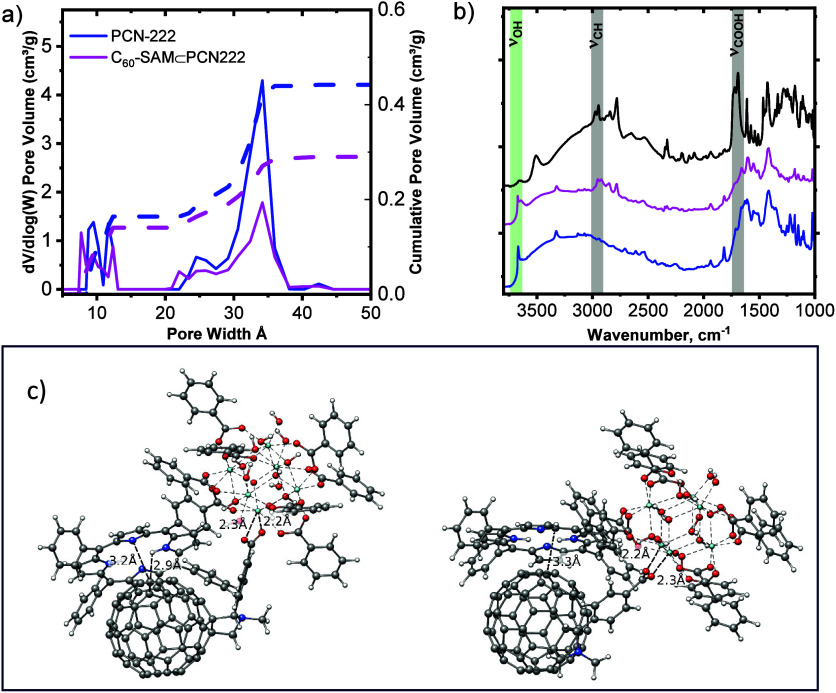
a) Pore size distribution and cumulative pore volume of PCN-222 and C_60_-SAM⊂PCN-222 extracted from N_2_ physisorption isotherms via a DFT model. b) DRIFTS spectra for (blue) PCN-222, (black) C_60_-SAM, and (pink) C_60_-SAM⊂PCN-222. Bands highlighted in gray correspond to C_60_-SAM-localized modes, while the green band is the terminal OH stretch of the Zr-oxo nodes in PCN-222. c) Geometry optimized structures depicting two different modes of C_60_-SAM carboxylate connectivity to the Zr-oxo cluster: bridging bidentate coordination (right) and nonbridging bidentate coordination (left).

Vibrational characterization via diffuse reflectance infrared Fourier transform spectroscopy (DRIFTS) indicates successful postsynthetic incorporation and coordination of C_60_-SAM into PCN-222. Notably, while other characteristic C_60_-SAM vibrational peaks are observed in the DRIFTS spectrum of the C_60_-SAM-appended framework (Figure 1b), the carboxylic acid peaks (i.e., the COOH stretch at 1691 cm^–1^ and OH stretch at 3506 cm^–1^) of the fullerene derivative guest are conspicuously absent, indicating full conversion to the carboxylate bidentate ligand attachment in PCN-222 after undergoing SALI. Furthermore, the red shift of the C–H stretching mode, attributed to the methyl appendage^12^ on the C_60_-SAM guest from 2951 cm^–1^ to 2978 cm^–1^, suggests CH_3_ perturbation upon confinement within the MOF. Changes in the MOF hydroxyl stretching mode region also provide some insight into the binding environment of the C_60_-SAM guest on the Zr-oxo clusters. The sharp OH stretch around 3688 cm^–1^, indicative of the terminal hydroxyl groups located on the zirconium-oxo cluster, appears along with a broad peak around 3643 cm^–1^ commonly associated with bridging hydroxyls. The persistence of the sharp OH peak in the spectrum of C_60_-SAM⊂PCN-222 is consistent with the subsaturation of available Zr-oxo coordination sites suggested by the other characterization methods (qNMR and N_2_ physisorption).

Computationally optimized structures show that upon attachment to the Zr-oxo cluster via carboxylate linkage, the fullerene derivative readily interacts with the porphyrin linkers. To explore these interactions, two large molecular cutouts—including a Zr-oxo cluster, a porphyrin linker, and a C_60_-SAM molecule—were generated starting from a published periodic structure.^13^ The first cutout models C_60_-SAM in a bidentate nonbridging coordination mode, where both carboxylate oxygens bind to a single Zr atom. The second cutout represents a bidentate bridging coordination mode in which each carboxylate oxygen binds to a different Zr atom. The optimized structures, shown in Figure 1c, exhibit short distances (2.9–3.3 Å) between the porphyrin linker and the C_60_ moiety, indicating significant π–π interactions. Relative reaction energies for the guest molecule binding to the MOF were calculated for both binding modes, evaluating r2SCAN-3c^14^ optimized structures with wB97X-V/def2-TZVPP^15^ single-point calculations. Solvation effects are included implicitly with SMD^16^ for dioxane. The nonbridging bidentate coordination is stabilized by 35.2 kcal/mol relative to the bridging mode. Overlaying the structures (Figure S9) reveals that the nonbridging binding mode centers the C_60_ over the porphyrin, strengthening the π–π contact compared to the bridging coordination. This is further quantified, through the calculation of a second reaction energy—without the C_60_ part of C_60_-SAM-COOH—where only benzoic acid (Ph-COOH) binds to the MOF. The difference between the first and the second energy reflects the stabilization of the complex through the interaction of the C_60_ atoms with the porphyrin. For the nonbridging mode, the complex is stabilized by 19.6 kcal/mol and for the bridging mode it is stabilized by 4.8 kcal/mol. The reaction equations and further descriptions are included in the Supporting Information.

Both UV–vis and PL titration measurements confirm the computational prediction and provide experimental evidence of the interaction between C_60_-SAM and the porphyrin linkers in the SALI-treated framework. The UV–vis spectrum of PCN-222 is dominated by the Soret band of the porphyrin linkers, with a peak maximum of 423 nm. Upon introduction of C_60_-SAM into the MOF, the intensity of the Soret band red-shifts and reduces (Figure 2). Like our previously reported C_60_⊂PCN222 MOF systems^6^ and other porphyrin-C_60_ complexes,^17–20^ this spectral change suggests perturbation of the porphyrin electronic structure due to through-space van der Waals interaction with the π system of the fullerene guest. A similar trend is observed in both DMF and 1,4-dioxane solvent environments. Binding constants are determined through UV–vis titration measurements by tracking changes in the Soret band as a function of the C_60_-SAM to porphyrin concentration ratio (shown as the inset in Figure 2). Analysis of the titration isotherms yielded *K*_assoc_ = 1.21 × 10^7^ M^–1^ for C_60_-SAM⊂PCN-222 in DMF, and *K*_assoc_ = 3.09 × 10^6^ M^–1^ in 1,4-dioxane. Figure 2 also shows the photoluminescence quenching behavior of PCN-222 upon titration with C_60_-SAM in DMF and 1,4-dioxane. Near complete quenching of the porphyrin emission is observed in each case at the highest concentrations of C_60_-SAM. Stern–Volmer analyses of the linear regimes of these quenching data (insets of Figure 2c and d) yield *K*_assoc_ = 1.47 × 10^7^ M^–1^ and 3.34 × 10^6^ M^–1^ in DMF and 1,4-dioxane, respectively. These values are comparable to the corresponding binding affinities obtained from the UV–vis titration measurements performed in these solvents and therefore suggest a static quenching mechanism within the concentration range. While the SALI introduction enforces a relatively fixed spatial position of the fullerene moiety in close proximity to the porphyin linker, the 4-fold difference in binding affinity observed in DMF compared to 1,4-dioxane reveals that the van der Waals interactions between the cluster-anchored fullerene and the porphyrin linker walls are strongly influenced by their solvation environment.

**Figure 2:**
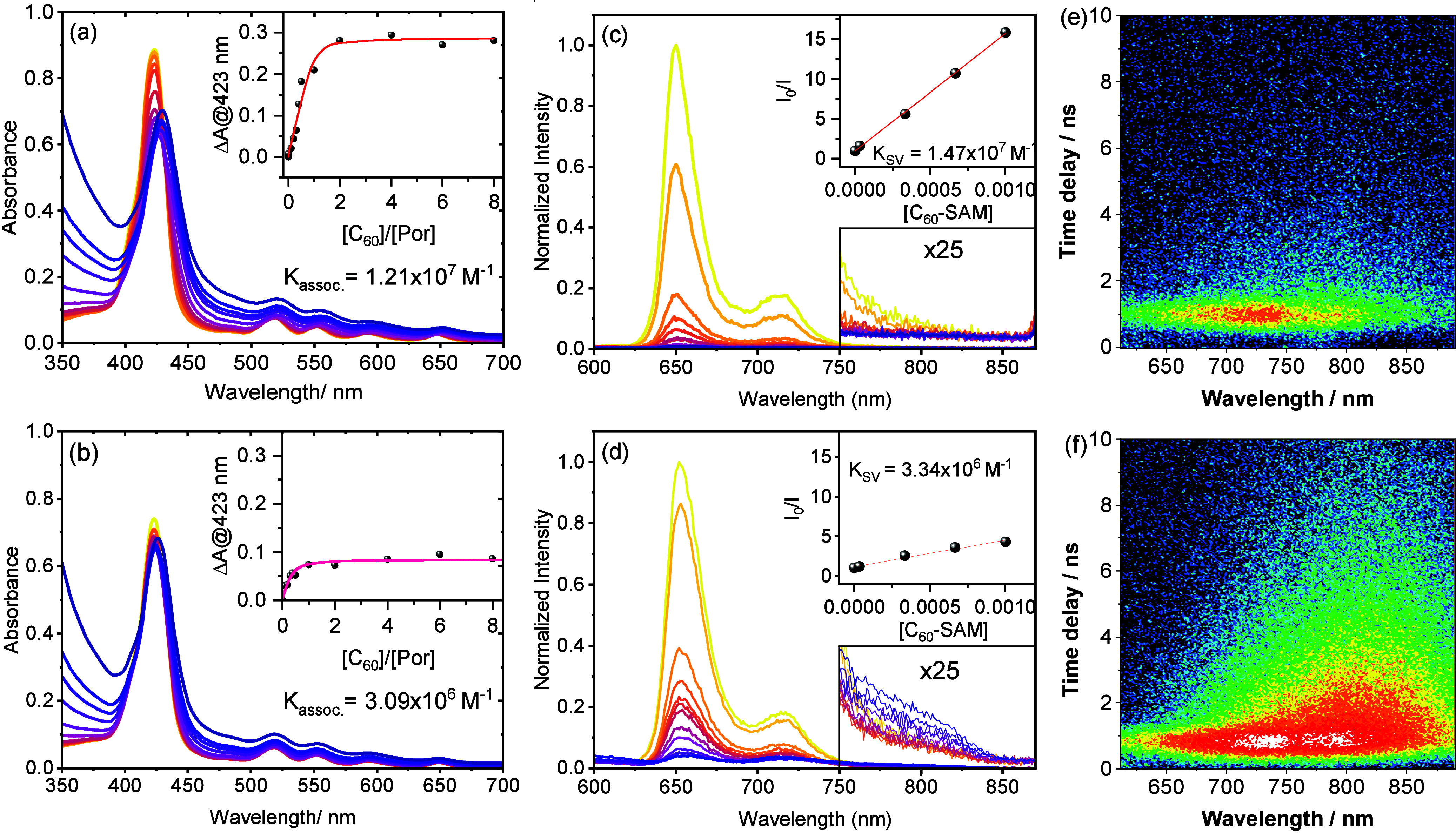
UV–vis titration isotherms collected as a function of relative fullerene/porphyrin concentration in (a) DMF and (b) 1,4-dioxane (insets: binding isotherms generated by monitoring absorbance difference at λ = 423 nm obtained for each fullerene concentration compared to PCN-222 with no C_60_-SAM added). Fluorescence titration measurements of C_60_-SAM⊂PCN-222 in (c) DMF and (d) 1,4-dioxane. (Upper insets: Stern–Volmer plots of *I*_0_/*I* as a function of the C_60_-SAM concentration). TRPL of C_60_-SAM⊂PCN-222 in (e) DMF and (f) 1,4-dioxane. Samples were excited at 410 nm, with plots showing spectrotemporal maps for the same amount of collection time (contour plotted on a log scale).

At the higher C_60_-SAM concentrations, steady state PL reveals a long wavelength emission feature around 810 nm (Figure 2d, inset), for C_60_-SAM⊂PCN-222 in 1,4-dioxane. We assign this feature as charge-transfer (CT) emission, and, consistent with the CT emission behavior in previously reported porphyrin–fullerene dyads, the radiative decay is not observed in polar media (DMF, in this case).^21–23^ In higher dielectric solvents, the CT state is stabilized in energy, making it more susceptible to nonradiative decay pathways due to the energy gap law.^24^ In low dielectric environments, the CT state remains at higher energy and undergoes weak radiative decay, resulting in observable CT emission.^21–23^

Time-resolved PL measurements provide initial insight into the dynamics of the CT excited state. As shown in Figure 2e, the initial spectra of C_60_-SAM⊂PCN-222 in 1,4-dioxane at early time delays contain convolved bands near 725 and 800 nm. The shorter wavelength feature, ascribed to C_60_-SAM singlet emission, decays within ∼1 ns, leaving the broad 800 nm PL band at longer times. This spectrum matches the CT emission feature in the steady-state spectrum shown in Figure 2d. In DMF, the amount of residual TR-PL near 800 nm remaining after the initial decay is much smaller than that observed in 1,4-dioxane (Figure 2f).

Transient absorption (TA) spectroscopy reveals the excited-state dynamics of C_60_-SAM⊂PCN-222 in polar vs nonpolar solvent environments. fs-TA spectral slices extracted over a 5 ns time delay window and the species associated spectra (SAS) derived through global fitting analysis for the C_60_-SAM⊂PCN-222 system in DMF and 1,4-dioxane are presented in Figure 3a,b. Upon 400 nm photoexcitation, C_60_-SAM⊂PCN-222 in each solvent undergoes instrument response-limited population of the S_1_ excited state as indicated by the appearance of its characteristic excited state absorption (ESA) peak at ∼480 nm along with the Soret and Q-band ground state bleach (GSB) features. Much like the analogous C_60_⊂PCN-222­(H_2_) MOF system,^6^ broad ESA bands around 600–800 nm (porphyrin cation) and 900–1200 nm (C_60_-SAM anion) observed within 1 ps indicate population of charge transfer (CT) excited states. The global fits of the data reveal multiexponential decay pathways consistent with our previous findings for C_60_⊂PCN-222­(H_2_). Following the local porphyrin singlet state population with subps decay lifetime, the target analyses revealed multiple intermediate state components with CT character: a shorter lifetime exciplex^23,25^ component that precedes longer lived formal CT excited states. The existence of two discernible CT species after initial exciplex formation was previously shown for C_60_ guests in PCN-222 ^6^ and may be a consequence of multiple structural and energy transfer relaxation pathways that accompany photoexcited charge separation in the guest–host framework with a range of fullerene distribution scenarios.

**Figure 3:**
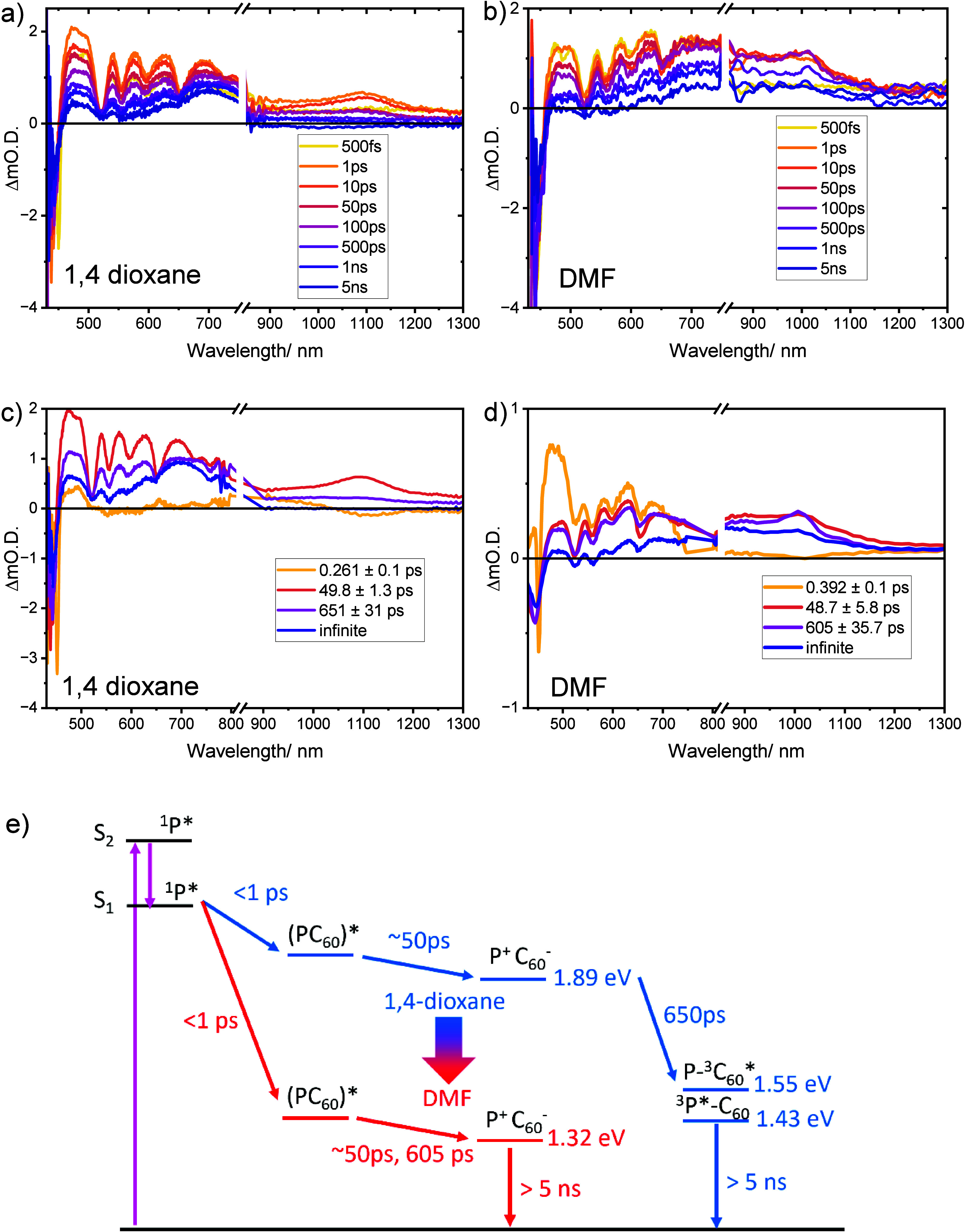
fs-TA spectral overlays for a) C_60_-SAM⊂PCN-222 in 1,4-dioxane and b) DMF; species associated spectra of C_60_-SAM⊂PCN-222 in c) 1,4-dioxane and d) in DMF, obtained from global fit to TA data using a sequential model; e) C_60_-SAM⊂PCN-222 schematic energy level diagram illustrating proposed charge recombination pathways in polar and nonpolar solvent environments (P = porphyrin linker, (PC_60_)* = exciplex).

We note that the ESA feature associated with the C_60_-SAM anion at ∼1100 nm in 1,4-dioxane decays within the 5 ns window to the porphyrin- and C_60_-SAM-localized triplet states as indicated by the ESA at ∼450 and ∼710 nm, respectively, evident at the longest time delays. While the peak position is consistent with C_60_ radical anion spectra generated through chemical reduction in nonpolar environments,^26^ broadening could indicate relatively close coupling with the porphyrin radical cation. In DMF, the C_60_-SAM anion peak appears at ∼1000 nm and persists beyond 5 ns, indicating long-lived charge-transfer states in this solvent environment. A shift of the observed radical anion band to higher energy could be the result of a lowering of its energy, which increases the transition energy to the excited level probed by TA. Free energy determinations through electrochemical measurements (see Supporting Information) predict a fully charge-separated state energy of 1.31 eV and Δ*G*_ET_ of −0.59 eV in DMF, which is well below the estimated energy of both the porphyrin triplet state (∼1.43 eV)^27^ and C_60_ triplet state (∼1.55 eV).^28^ In 1,4-dioxane, the estimated charge-separated state energy (1.89 eV) remains above those of the porphyrin and C_60_ triplet states, consistent with the assignment that the CT state decays through the porphyrin and C_60_ triplet state channels in nonpolar solvents. The excited-state decay pathways in the two solvent environments are summarized in the energy level diagrams shown in Figure 3.

Loading the PCN-222 framework with varying amounts of C_60_-SAM alters the kinetics of charge recombination in DMF (Figure 4a). With increased loading, the initial decay time of the C_60_ anion feature is elongated, leaving a residual signal beyond 5 ns. TA at longer time scales (Figure 4b) reveals the persistence of C_60_ anion absorption in C_60_-SAM⊂PCN-222 and its dependence on loading concentration. The slower component appears to be biexponential, having an initial decay of around 100 ns, followed by a decay of around 1 μs. These secondary decay components grow with increasing C_60_-SAM loading (Table S4). Upon global fitting (Figure 4c), the 80–150 ns component spectrum roughly matches that of the 5 ns decay obtained from the fs-TA measurements, but the slowest decay spectrum exhibits a blue-shifted primary peak that bears resemblance to C_60_ dimer anion spectra previously measured and calculated.^29^ The loading dependence suggests that C_60_ aggregation may be responsible for providing the driving force that permits observable long-lived charge-separated states, as opposed to proximal or tightly bound CT that may recombine quickly to the triplet.^30^ To assess whether the framework geometry permits such aggregation, periodic molecular dynamic (MD) simulations were performed using the mcGFN-FF method.^31,32^

**Figure 4:**
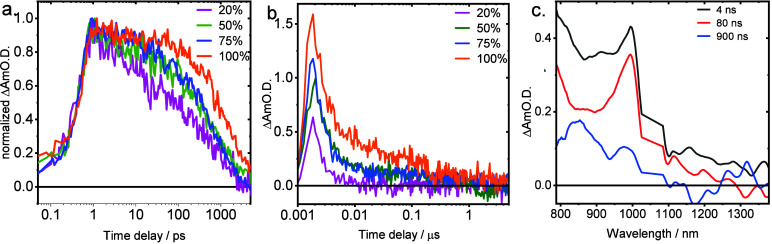
Kinetic slices at 1000 nm of C_60_-SAM⊂PCN-222 with 20%, 50%, 75%, 100% fullerene loading (see text for details) on (a) fs-ns and (b) ns-μs time scales. For (b), the instrument response is roughly 1 ns, and time zero is offset to 2 ns for clarity on a logarithmic scale. (c) Species associated spectra of the longest lived species in a sequential model for the 100% loaded sample. Four ns component is reduced by 3× for comparison with other components. Scatter signals near 1050 nm are removed.

As illustrated in Figure 5, the simulations reveal that only specific relative binding configurations of C_60_-SAM molecules allow persistent short intermolecular separations. In favorable arrangements, the C_60_ moieties remain within approximately 3–4 Å over the majority of the simulation time, enabling substantial intermolecular interactions while remaining coordinated to the framework. In contrast, alternative binding configurations lead to significantly larger separations and do not support aggregation. These results indicate that aggregation is not a generic feature of the system but arises only for particular relative binding sites. At full (100%) loading, at least one pair of C_60_ molecules is within 4.2 Å of each other during the entire 50 ps simulation (Figure S10). Consequently, increased molecular loading is expected to enhance the statistical likelihood of such aggregation-permitting configurations, providing a plausible microscopic origin for the observed long-lived CT states. Additional simulations and structural illustrations are shown in the Supporting Information.

**Figure 5:**
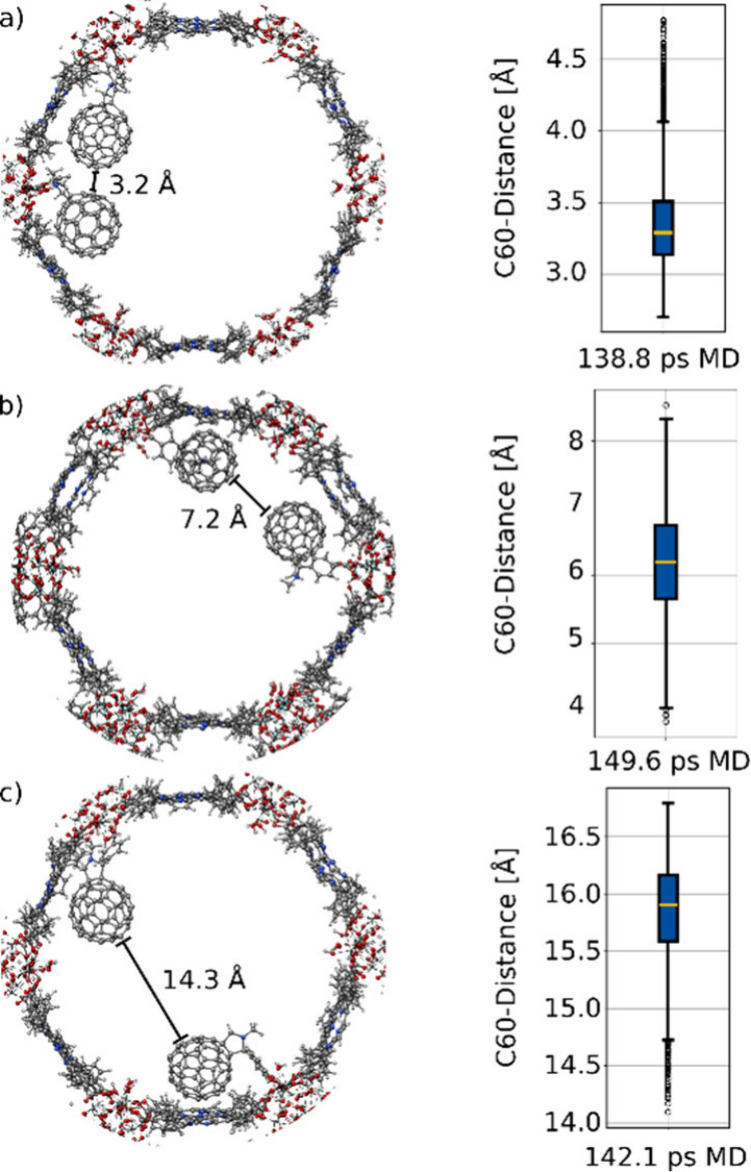
Illustration of the periodic system used for the MD simulation showing two C_60_-SAM-COOH molecules within the large hexagonal pore, together with a boxplot of the distance between the C_60_ moieties for molecules bound to (a) adjacent Zr-oxo clusters, (b) Zr-oxo clusters separated by one empty cluster, and (c) Zr-oxo clusters on opposite sites.

Taken as a whole, the differences in behavior observed between polar vs nonpolar solvents can be attributed to the C_60_-SAM acceptor species located in the large pores of the MOF. In the previously reported C_60_⊂PCN-222­(H_2_) system, the confinement of C_60_ in the triangular pore prevented solvent from accessing the C_60_-porphyrin interface, and thus the charge transfer and subsequent decay pathways were not influenced by the dielectric environment. When located in the large pores and tethered close to the porphyrin linker sites via SALI, the C_60_-SAM acceptors are solvent accessible, and the resulting energy stabilization in polar solvent leads to long CT state lifetimes. Furthermore, the ability to select the charge acceptor pore location and leverage the associated dielectric and steric environments permits a high degree of control over the subsequent photophysics. The current platform shows promise for expansion to include measures of bulk (photo)­conductivity and the introduction of catalytically active metallic species in the porphyrins for selective chemical transformations.

## Methods

PCN-222 MOF nanoparticles were synthesized following literature precedent^9^ with previously reported modifications and washing steps.^6^ C_60_-SAM was subsequently loaded into the PCN-222 framework using the solvent-assisted ligand insertion (SALI) method.^9^ 10 mL toluene solutions with different C_60_-COOH concentrations were introduced to a 10 mL DMF suspension of PCN-222­(H_2_) followed by incubation at 60 °C for 24 h. This procedure was used to generate C_60_-SAM⊂PCN-222­(H_2_) with 0.2:1, 0.5:1, 0.75:1 and 4:1 C_60_-SAM:porphyrin linker stoichiometric ratios. Each 20 mL of C_60_-SAM⊂PCN-222 nanoparticle suspension was centrifuged at 10,000 rpm for 10 min followed by decantation and addition of 20 mL of 1:1 DMF:toluene fresh solvent. This centrifuge step was repeated three times followed by resuspension in 20 mL of DMF.

Powder X-ray diffraction (PXRD) measurements were collected using a Rigaku MiniFlex 6G instrument running at 40 kV and 15 mA (600 W). The amount of C_60_-SAM loaded into the MOF was estimated using a previously reported weighing method applied to similar fullerene derivative-loaded MOF systems^7,8^ (see Supporting Information for details) and quantitative NMR. NMR experiments were carried out on a Bruker Avance III HD 500 MHz equipped with a broadband probe. Relaxation delay was set to 10 s to ensure the full relaxation of the ^1^H signals in the spectrum. N_2_ physisorption isotherms were collected at 77 K using a Micromeritics ASAP 2020 with 45 s equilibration time in the *p*/*p*_0_ range of 0 to 0.001 and 10 s for *p*/*p*_0_ > 0.001. Further details describing the quantitative analysis procedures and pore volume analysis are provided in the Supporting Information. Diffuse reflectance infrared Fourier transform spectroscopy (DRIFTS) data averaged over 2048 scans with a 4 cm^–1^ resolution were obtained by using a Nicolet FTIR with an MCT-A detector. UV–visible absorption spectra were collected using a Cary 5000 UV–vis spectrophotometer. Liquid suspension samples were measured in a quartz cell with a 1 cm optical path. The fluorescence data were collected using a Horiba Fluorolog-3 spectrofluorometer using λ = 421 nm excitation wavelength. C_60_-SAM⊂PCN-222­(H_2_) suspension samples were prepared as described previously^6^ with a range of C_60_-SAM concentrations (0.01–16 mol equivalents) for both UV–vis titration measurements and photoluminescence (PL) quenching studies.

Time-resolved photoluminescence data were collected using a Hamamatsu Streak Camera (300–900 nm, C10910-04) with an NKT supercontinuum fiber laser (SuperK EXU-6-PP) routed to an acousto-optic tunable filter (SuperK Select). The samples were photoexcited at 410 nm with a repetition rate of approximately 1 MHz.

Femtosecond transient absorption (fs-TA) measurements were performed on MOF suspensions in a 2 mm path length quartz cuvette. Data were collected using a Coherent Libra Ti:sapphire laser operating at 800 nm and 1 kHz. Femtosecond excitation pulses (∼150 fs) at 400 nm were generated using a Light Conversion TOPAS-C optical parametric amplifier to excite near the maximum of the porphyrin Soret absorption band. The pump pulse was 100 nJ with a pump beam diameter of ∼300 μm for the sample using a ThorLabs beam profiler. In an Ultrafast Systems Helios spectrometer, a visible probe light (450–800 nm) was generated by focusing a fraction of the 800 nm fundamental into a 1 mm sapphire crystal, while a near-infrared supercontinuum probe light was generated in a 10 mm sapphire crystal. The Helios delay stage generated a delay of up to 5 ns. NsTA uses the same excitation beam with the probe provided by an electronically delayed supercontinuum beam (Ultrafast Systems EOS, 800–1600 nm). The time resolution is roughly 1 ns. Two dimensional (2D) maps were processed in Surface Xplorer by averaging three spectra followed by background and scattering light subtraction, chirp correction, and extraction of single wavelength kinetic slices. The processed data were subsequently analyzed in MATLAB using a custom global fitting routine to obtain decay kinetics and species associated spectra (SAS).

The initial model systems were generated using Avogadro2.^33,34^ The cutouts were then optimized with r2SCAN-3c,^14^ including solvation effects with SMD^16^ for dioxane. Methyl and hydrogen atoms added to saturate dangling bonds were preoptimized and then held fixed during the optimization, preserving the structural features of the periodic framework. All electronic structure calculations were performed with ORCA 6.0.1.^35–38^ The MD simulations were performed using xtb version 6.7.1.^39,40^ Illustrations of the model systems were created with chimera.^41,42^

## Supplementary Material



## Notes

Materials, powder XRD, fullerene loading estimation, quantitative NMR, N_2_ physisorption isotherms, TRPL spectroscopy results, TA global fit amplitude and lifetime data, electrochemical methods description, Gibbs free energy calculations, additional fs-TA and ns-TA spectroscopy results, computational details, additional computational results (PDF)
