# Supplementary material for: Pore-Selective Fullerene Loading in a Porphyrin-Based Metal–Organic Framework Controls Photoinduced Charge-Transfer Dynamics
Source: J Phys Chem Lett. 2026 Apr 9;17(16):4811–9. doi: 10.1021/acs.jpclett.6c00574 (PMC13112442; doi:10.1021/acs.jpclett.6c00574)
Supplement: Supplementary file 1 [file jz6c00574_si_001.pdf]

Supporting Information for Publication:

Pore-Selective Fullerene Loading in a Porphyrin-Based Metal-Organic Framework Controls Photoinduced Charge-Transfer Dynamics.

Alison Arissa[1], Thomas Rose[2], Noémi Leick[4], Pavel Kucheryavy[1], Junjie Ouyang[1], Huixin He[1], Stefan Grimme[3], Justin C. Johnson*[4], Jenny V. Lockard*[1]

1. Department of Chemistry, Rutgers University-Newark, Newark, New Jersey 07102, United States
2. Max-Planck-Institut für Kohlenforschung, D-45470 Mülheim an der Ruhr, Germany
3. Mulliken Center for Theoretical Chemistry, Clausius-Institut für Physikalische und Theoretische Chemie, Rheinische Friedrich-Wilhelms Universität Bonn, Bonn 53115, Germany
4. National Laboratory of the Rockies, 15013 Denver West Parkway, Golden, Colorado 80401, United States

*Email: justin.johnson@nlr.gov , jlockard@newark.rutgers.edu

Materials

Meso-tetracarboxyphenylporphyrin (TCPP), and tetraphenylporphyrin (TPP) were synthesized using literature precedent.[1-3] Zirconyl chloride cctahydrate (99 % purity) was purchased from Sigma Aldrich. N'N dimethylformamide (DMF), and toluene were purchased from Millipore Sigma. Dichloroacetic acid (DCA)

(97 %) was purchased from TCI chemicals. 4-(1',5'-Dihydro-1'-methyl-2'H-[5,6]fullereno-C60-Ih-[1,9-c]-pyrrol-2'-yl)benzoic acid, (C_{60}-SAM) was purchased from Luminescence Technology Corp.

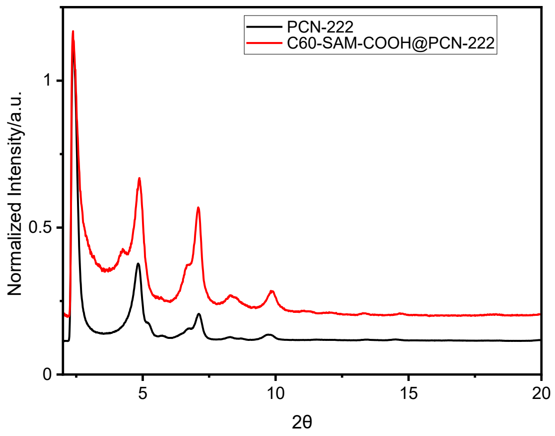

Figure S1. PXRD patterns obtained for PCN-222 before and after introduction of C_{60}-SAM using the SALI method.

Method for estimating fullerene loading amount

In this procedure, adapted from a previous reported method, [4-5] 10 drops of sulfuric acid were added to ~5mg of C_{60}-SAM⊂PCN-222 to digest the MOF portion of the sample. The suspension was sonicated until fully dispersed followed by centrifugation at 10,000 RPM for 5 minutes. The insoluble C_{60}-SAM brown precipitate was subsequently resuspended in 1 mL of water and sonicated until fully dispersed, and then centrifuged down using 10,000 RPM for 5 minutes. This process was repeated two more times. Then, after decanting the last aliquot of water, acetone was added to the brown pellet and the mixture was sonicated to suspension. This process was repeated 3x until finally the brown pellet of C_{60}-SAM was dried in a vacuum oven at 80°C overnight.

Quantitative NMR experiments

Quantitative NMR experiments were carried out on 500 MHz Bruker Avance-III HD NMR spectrometer equipped with broad band probe with Z-gradient. Relaxation delay was set to 10 s to ensure full relaxation of all resonances. MOF samples were weighed out mixed with 0.75 mL DMSO-d_6 and 100 μL of 98% sulfuric acid and placed in the oven at 90° C for 1 hour. After that an internal standard (1,3,5-trimethoxybenzene, or maleic acid) and toluene-d8 were added and ^{1}H NMR was recorded. During digestion process, besides the MOF decomposition, the following processes occur for the solvent and for 1,3,5-thimethoxybenzene (TMB) internal standard:

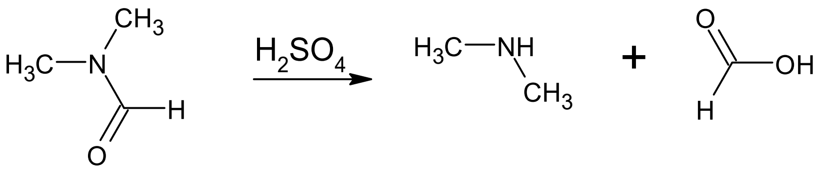

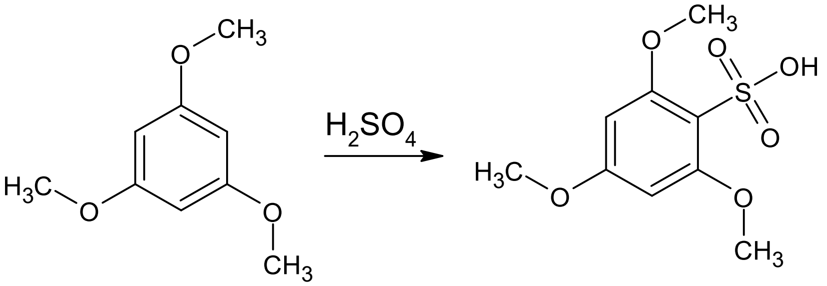

Calculations were performed according to the following equations:

Mass of the sample:

$$m_{Sample} = m_{MOF} + m_{DMF} + m_{Me_2NH} + m_{HCOOH} \quad (1)$$

Equations for calculation of the amount of sample:

$$n_{Sample} = \frac{I_S/n\mathrm{H}_S}{I_{IS}/n\mathrm{H}_{IS}} \times n_{IS} \quad (2)$$

n_{Sample} – amount of sample

n_{IS} – amount of internal standard

I_S – Intensity of the sample signal

I_{IS} – Intensity of the internal standard signal

$n\mathrm{H}_S$ – number of protons in sample

nH_{IS} – number of protons in internal standard

$$m_{TCPP} = 786.71 \times n_{TCPP} \quad (3)$$

$$m_{C60SAM} = 897.84 \times n_{C60SAM} \quad (4)$$

$$m_{DMF} = 73.095 \times n_{DMF} \quad (5)$$

$$m_{Me_2NH} = 45.084 \times n_{Me_2NH} \quad (6)$$

$$m_{HCCOH} = 46.015 \times n_{HCOOH} \quad (7)$$

Amounts of all components of digested sample can be derived from integral values and qNMR analysis using equation 2.

Molecular weight of residual cluster (should be 401.62 for PCN-222):

$$MW_{Cluster} = \frac{m_{Sample} - m_{TCPP} - m_{DMF} - m_{Me_2NH} - m_{HCOOH}}{n_{TCPP}} \quad (8)$$

$$MW_{Cluster} = \frac{m_{Sample} - m_{TCPP} - m_{C60SAM} - m_{DMF} - m_{Me_2NH} - m_{HCOOH}}{n_{TCPP}} \quad (9)$$

Ratio of the TCPP to C60SAM:

$$\frac{n_{TCPP}}{n_{C60SAM}} = \frac{I_{TCPP}}{I_{C60SAM}} \quad (10)$$

Tables S1 – 3. qNMR analysis of C_{60}SAM-PCN222 samples

					^{1}H		
	MW (g/mol)	m, mg	n (mmols)	δ, ppm	I	nH	n (mmols)
Standard	168.19	2.25	0.013378	3.61	8.86	9	
TCPP	786.79	1.87	0.002377	8.57	0.58	8	0.000985
TCPP	786.79	1.87	0.002377	8.72	0.555	8	0.000943
TCPP	786.79	1.87	0.002377	8.66	0.555	8	0.000943
C60SAM	896.84	1.87	0.002085	2.43	0.04	1	0.000544
C60SAM	896.84	1.87	0.002085	3.31	0.04	1	0.000544
DMF	73.09	1.87	0.025585	2.82	0.17	3	0.00077
DMF	73.09	1.87	0.025585	2.66	0.22	3	0.000997
Me2N	45.08	1.87	0.041482	2.42	0.63	6	0.001427
HCOOH	46.02	1.87	0.040635	8.1	0.15	1	0.002038

					^{1}H		
	MW (g/mol)	m, mg	n (mmols)	δ, ppm	I	nH	n (mmols)
Standard	116.72	2.27	0.019448	6.16	2	2	
TCPP	786.79	7.68	0.009761	8.57	1.52	8	0.003695

TCPP	786.79	7.68	0.009761	8.78	1.475	8	0.003586
TCPP	786.79	7.68	0.009761	8.76	1.475	8	0.003586
C60SAM	896.84	7.68	0.008563	2.43	0.09	1	0.00175
C60SAM	896.84	7.68	0.008563	3.31	0.09	1	0.00175
DMF	73.09	7.68	0.105076	2.82	1.6	3	0.010372
DMF	73.09	7.68	0.105076	2.66	1.64	3	0.010632
Me2N	45.08	7.68	0.170364	2.42	3.02	6	0.009789
HCOOH	46.02	7.68	0.166884	8.1	0.54	1	0.010502

					^{1}H		
	MW (g/mol)	m, mg	n (mmols)	δ, ppm	I	nH	n (mmols)
Standard	168.19	4.54	0.026993	5.96	3	3	
TCPP	786.79	5.64	0.007168	8.71	0.66	8	0.002227
TCPP	786.79	5.64	0.007168	8.61	0.67	8	0.002261
TCPP	786.79	5.64	0.007168	8.55	0.67	8	0.002261
C60SAM	896.84	5.64	0.006289	5.55	0.05	1	0.00135
C60SAM	896.84	5.64	0.006289	5.37	0.05	1	0.00135
DMF	73.09	5.64	0.077165	8.76	0.86	3	0.007738
DMF	73.09	5.64	0.077165	8.61	0.89	3	0.008008
Me2N	45.08	5.64	0.125111	2.46	1.74	6	0.007828
HCOOH	46.02	5.64	0.122555	8.15	0.63	1	0.017006

Based on the qNMR analysis ratio of C_{60}SAM to linker (TCPP) 1 ± 0.55.

Ratio of DMF to TCPP to C_{60}SAM is 16.51:1.83:1.

The residual molecular weight of the Zr cluster is 847.62 ± 3.61 which can be attributed to Hf impurity.

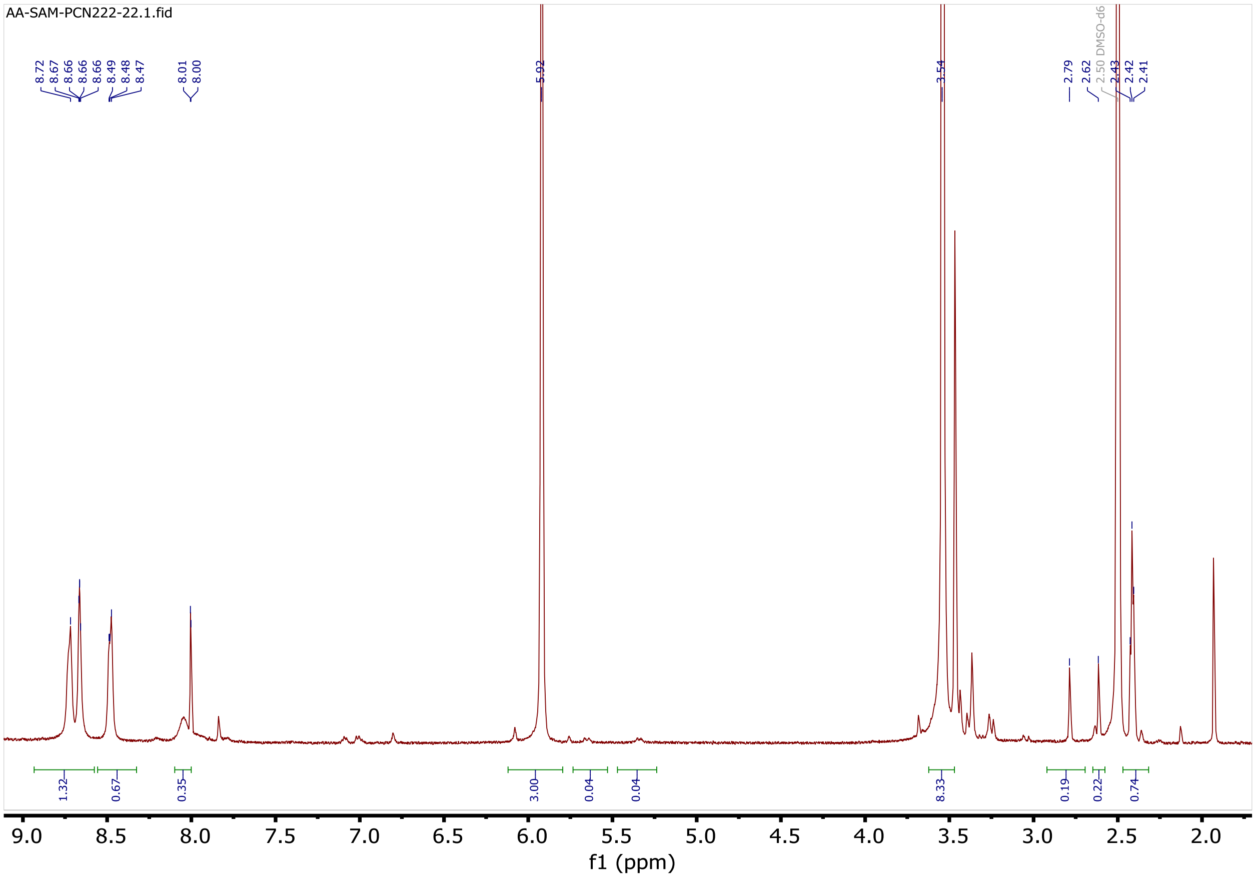

Figure S2. qNMR of C_{60}-SAM⊂PCN-222(H_2)

N_2 Physisorption

The samples were first degassed under $1x10^{-5}$ Torr vacuum at room temperature for 15 h and then heated to 120 °C over 30 min and held at 120° C for 2.5 h prior to surface area and pore size distribution measurements. Measurements were collected using a home-built temperature programmed desorption setup equipped with a residual gas analyzer (RGA) with mass-to-charge ratio sensitivity from 1-200 amu. RGA confirmed complete removal of remaining solvent and water molecules that would potentially obstructing N_2 binding sites. N_2 physisorption isotherms were collected at 77 K using a Micromeritics ASAP 2020 with 45 s equilibration time in the p/p_0 range of 0 to 0.001 and 10 s for $p/p_0 > 0.001$. The specific surface areas were extracted from the isotherm data using the Brunauer-Emmett-Teller (BET) model in the p/p_0 range of 10^{-5} to

0.1,respecting the Rouquerol criterion.[6] Pore size distributions and cumulative pore volume of each sample were modeled in the range of $15x10^{-5} < p/p_0 < 0.95$ using the"N_2 @77K – oxide cylindrical pores Tarazona" commercially available DFT model for cylindrical geometries available in the Micromeritics software.

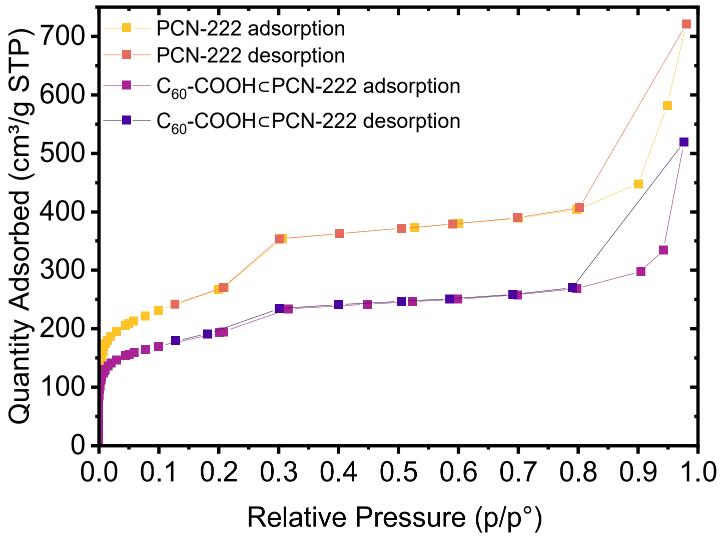

Figure S3. Nitrogen physisorption isotherms of PCN-222 and C_{60}-SAM⊂PCN-222(H_2)

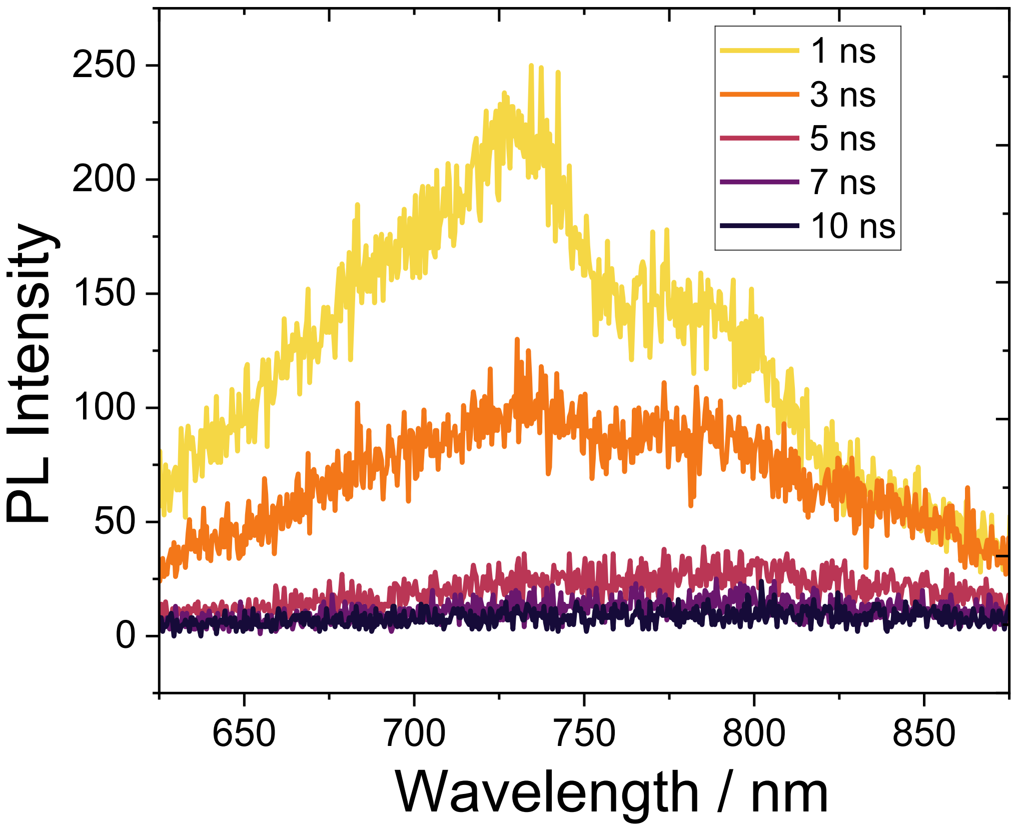

Figure S4. TRPL spectral slices of C_{60}-SAM⊂PCN-222(H_2) in DMF

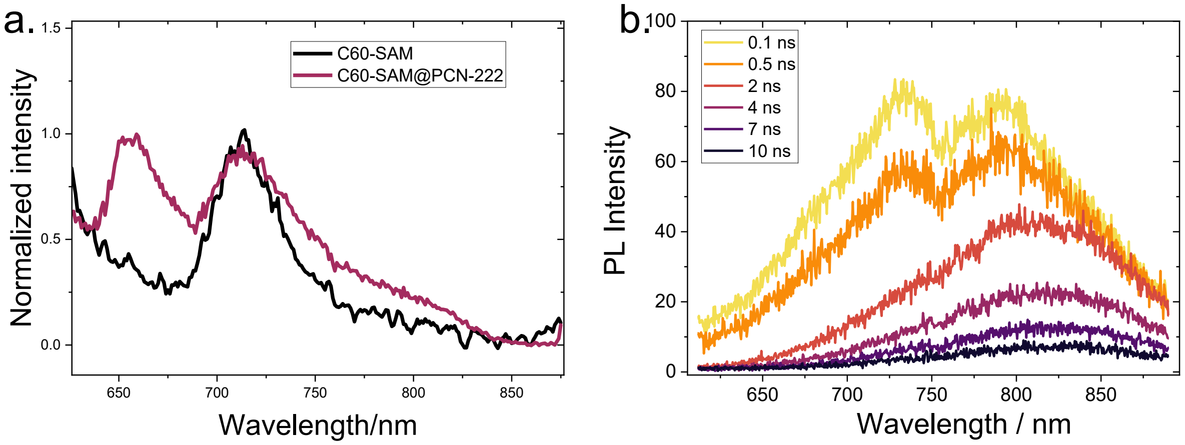

Figure S5. (a) steady state emission spectra and (b) TR-PL spectral slices of C_{60}-SAM⊂PCN-222 in 1,4-dioxane

Table S4. Amplitudes and time constants for three-exponential global fit to ns-μs transient absorption data for C_{60}-SAM⊂PCN-222 in DMF. A_3/τ_3 represents the blue-shifted spectral features near 850 nm, which could not be distinguished for 50% and 20% loading.

loading	A_1	A_2	A_3	τ_1	τ_2	τ_3
100%	1.3	0.36	0.18	1.8 ns	80 ns	868 ns
75%	1.0	0.16	0.16	1.4 ns	75 ns	2.2 μs
50%	0.62	0.10	--	1.2 ns	131 ns	--
20%	0.45	0.09	--	1.4 ns	152 ns	--

Electrochemical methods

Cyclic voltammetry (CV) measurements were carried out using a CH Instruments (CHI 760C) electrochemical workstation in a three-electrode configuration. A glassy carbon plate electrode (10 mm × 10 mm, geometric area = 1.0 cm^2) modified with a C_{60}-SAM film was used as the working electrode, with a platinum plate as the counter electrode and an Ag/AgCl electrode as a pseudo-reference electrode. Ferrocene was used as an internal standard (2.5 mM), and all potentials reported in this work were referenced to the Fc/Fc^+ redox couple. The electrolyte was 0.1 M tetrabutylammonium hexafluorophosphate ($TBAPF_6$) in dichloromethane (DCM), which was degassed with nitrogen prior to measurements.

C_{60}-SAM films were prepared by dispersing the C_{60}-SAM in isopropanol via ultrasonication (10 mg mL^{-1}), followed by drop-casting 20 μL of the suspension onto the glassy carbon electrode and drying at 70 °C for 30 min. CV measurements of as-prepared C_{60}-SAM films were performed over a potential window from -0.185 to -1.685 V vs Fc/Fc^+. While the CV measurement of TPP was carried out with TPP dissolved in the electrolyte solution at a concentration of 0.25 mM, with the potential window ranging from -0.285 to +1.015 V vs Fc/Fc^+. All the CVs were recorded at scan rate 150 mV s^{-1}.

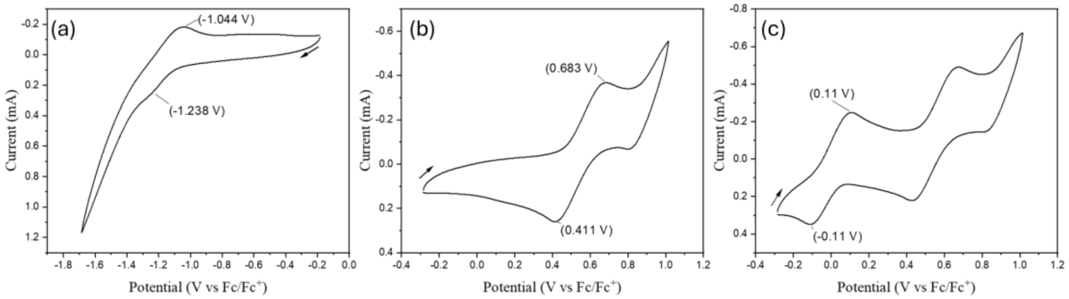

Figure S6. Cyclic voltammograms of (a) C_{60}-SAM film, (b) TPP (0.26 mM), and (c) ferrocene (2.5 mM) recorded in 0.1 M $TBAPF_6$DCM using an Ag/AgCl pseudo-reference electrode. All potentials in the main text are referenced to Fc/Fc^+.

Gibbs Free Energy Calculation for C_{60}-SAM⊂PCN-222

The Gibbs free energy change of photoinduced electron transfer in DMF is -0.589 eV and in 1,4-dioxane is -0.0191 eV solved by the Gibbs free energy change with Born correction:[7]

$$\Delta G_{PET} = \mathrm{F}\left(E_{\frac{1}{2}}^{ox}(D) - E_{\frac{1}{2}}^{red}(A)\right) + \mathcal{W} - \mathcal{E}0 - 0 + \Delta G_S \qquad (11)$$

F is Faraday constant, $\mathcal{E}_{0\text{–}0}$ is the lowest excited singlet state energy of the donor (1.91 eV), $E_{1/2}^{ox}(D)$ is the one electron oxidation potential of the donor, $E_{1/2}^{red}(A)$ is the one electron reduction potential of the acceptor. The following are two electrostatic correction terms:

Born Correction (ΔGS), Electrostatic work ($\mathcal{W}$) where:

$$\Delta GS = \frac{nq^2}{8\pi\varepsilon_0}\left(\frac{2z_D+n}{r_D}\left(\frac{1}{\varepsilon_r} - \frac{1}{\varepsilon_D}\right) - \frac{2z_A-n}{r_A}\left(\frac{1}{\varepsilon_R} - \frac{1}{\varepsilon_A}\right)\right) \qquad (12)$$

$$\mathcal{W} = \frac{q^2 n(z_A - z_D - n)}{4\pi\varepsilon_0\varepsilon_r R_{DA}} \qquad (13)$$

Where n is number of transferred electrons, q is the electron charge, z_D is the initial charge of the donor, z_A is the starting charge of the acceptor, ε_0 is permittivity of free space, ε_R is the dielectric constant of the solvent where PET takes place, ε_D is the dielectric constant of the solvent of the donor where redox potentials were measured, ε_A is the dielectric constant of the solvent the acceptor where redox potentials were measured. Using an optimized segment of C_{60}-SAM⊂PCN-222, the distance between the center of the donor and acceptor was measured (R_{DA}), along with the radius of the donor and acceptor (r_D and r_A, respectively).

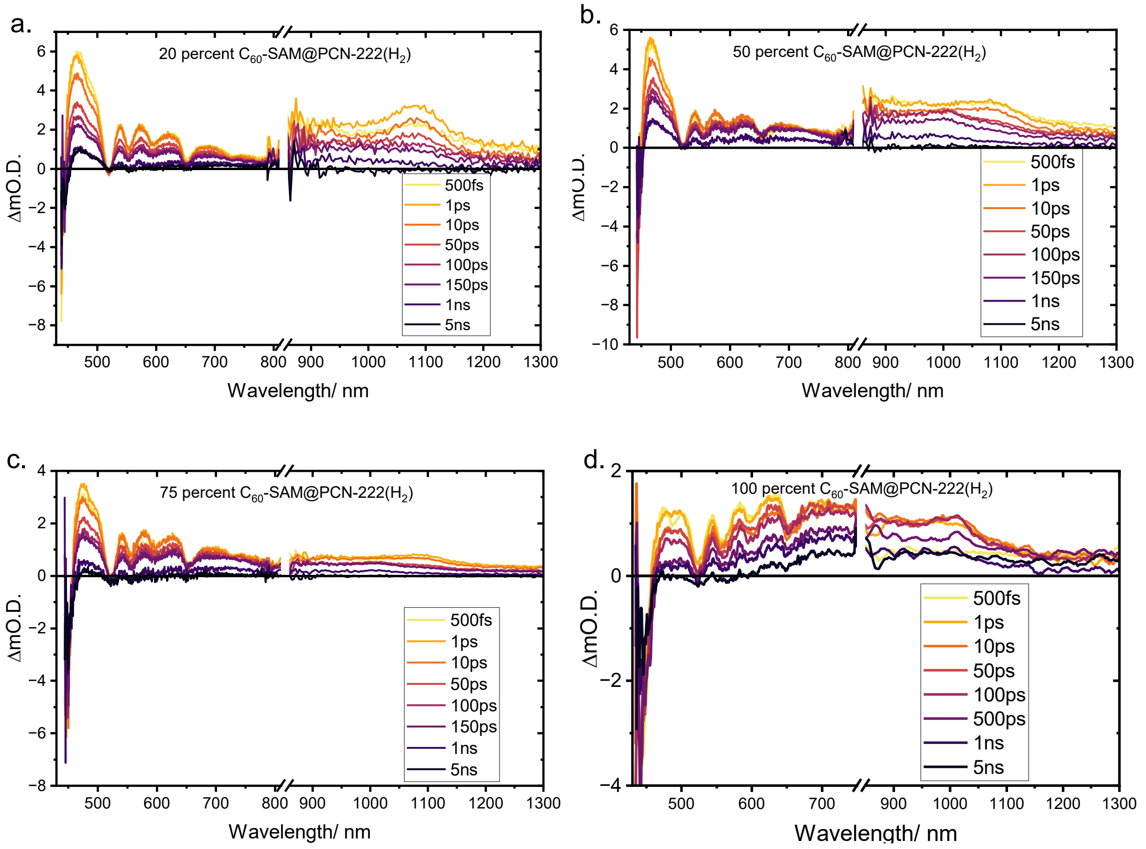

Figure S7. fs-TA spectral slices of C_{60}-SAM⊂PCN-222 in DMF with a.) 20 %, b.) 50 % c.) 75 %, and d.) 100 % C_{60}-SAM loading

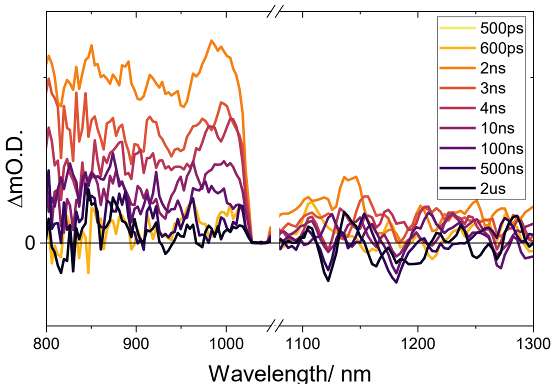

Figure S8. ns-TA spectral slices C_{60}-SAM⊂PCN-222 in DMF with 100% loading

Computational Details

Two reaction energies were calculated to estimate how much the interaction between C_{60} and porphyrin contributes to the binding energy of C_{60}-SAM-COOH to the MOF (PCN222). The first reaction is the C_{60}-SAM-COOH binding to the MOF:

$$C_{60}\text{-SAM-COOH} + \text{MOF} \longrightarrow C_{60}\text{-SAM-COOH}\subset\text{MOF} + 2H_2O$$

The reaction energy for the non-bridging mode is –21.5 kcal/mol and 13.8 kcal/mol for the bridging mode.

The second reaction is benzoic acid (Ph-COOH) binding to the MOF:

$$\text{Ph-COOH} + \text{MOF} \longrightarrow \text{Ph-COOH}\subset\text{MOF} + 2H_2O$$

The reaction energy for the non-bridging mode is –1.8 kcal/mol and 18.5 kcal/mol for the bridging mode. The phenyl ring of the Ph-COOH for the second reaction is fixed in the position of the optimized complex from the first reaction. This allows drawing conclusions from the

reaction energies with respect to the interaction between the C_{60} and the porphyrin, since the missing C_{60} (and part of the linker) is the only difference between the two reactions.

The overlay of the bidentate and bridging mode of C_{60}-SAM in Figure S9, shows that the bidentate mode allows for more overlap between the C_{60} atoms and the porphyrin linker.

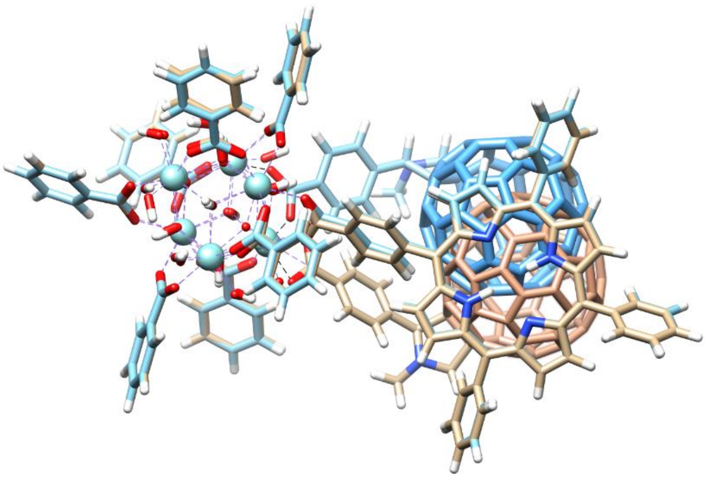

Figure S9. Superposition of optimized cutouts of C_{60}-SAM-COOH bound to a Zr-oxo cluster. The structure highlighted in blue is bound in a bidentate bridging coordination mode and the structure highlighted in light brown is bound in a bidentate non-bridging coordination mode.

Periodic molecular dynamics simulations were conducted using the mcGFN-FF force field to evaluate the dependence of intermolecular C_{60}–C_{60} distances on relative binding configurations within the framework. A unit cell containing two C_{60}-SAM molecules in a single large pore was employed to systematically probe aggregation behavior as a function of binding site geometry. Simulations were performed for three in-plane binding configurations, in which both molecules were coordinated within the same porphyrin layer at Zr-oxo clusters classified as *ortho*, *meta*, or

para relative positions, by analogy to benzene substitution patterns within the hexagonal pore (six Zr-oxo clusters per pore layer).

The start and end geometry from the MD simulation with one C_{60}-SAM-COOH per porphyrin (100% loading) are depicted in Figure S10, alongside the distances of the C_{60} atoms from the six C_{60}-SAM-COOH molecules in the same porphyrin layer. The smallest distance between all neighboring C_{60} parts was calculated for each timestep of the simulation and then maximum of these smallest distances was calculated to be 4.2 Å.

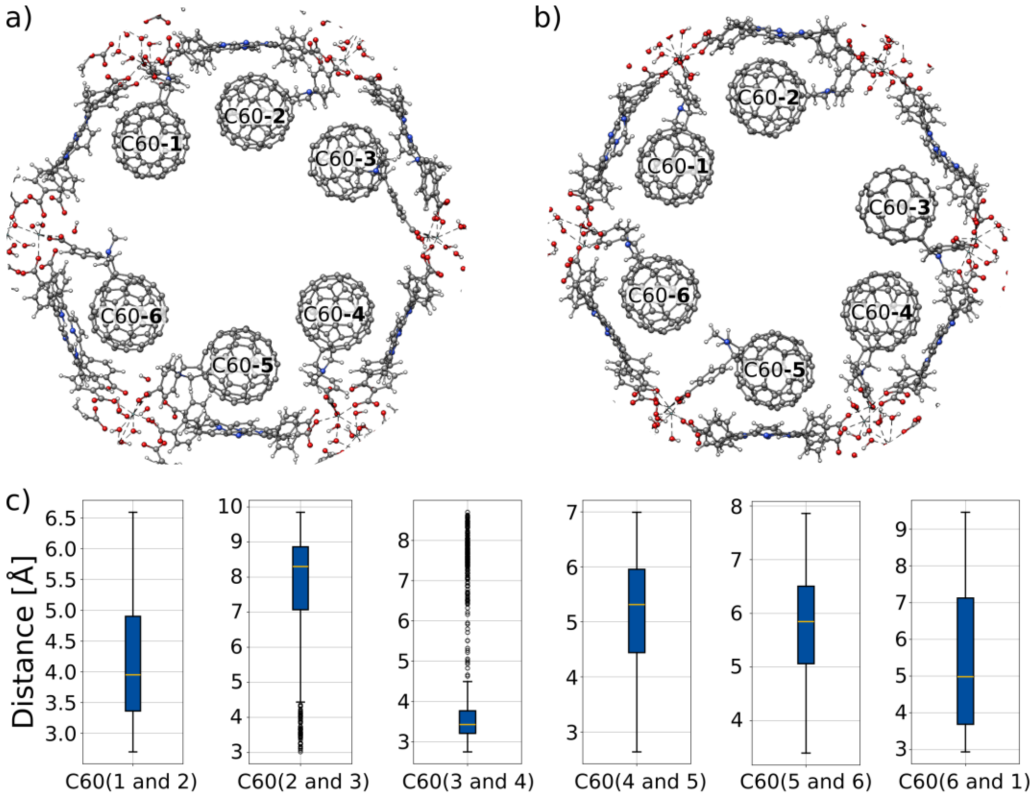

Figure S10. a) Starting positions for the periodic MD simulation of C60-SAM-COOH molecules within the large pore of PCN222(H_2). b) Illustration of the final structure of the MD simulation. c) Boxplots of distances between neighboring C60 moieties over the duration of the simulation.

In addition to the simulations presented in the main manuscript, two simulations were performed with two C_{60}-SAM above each other (along the direction of the hexagonal pore). In Figure S11, the start and end geometries from the simulations are depicted, as well as the boxplots for the C_{60} distances for both systems. With both C_{60}-SAM molecules bound to the same Zr-oxo cluster, the two molecules move away from each other. Additionally, the C_{60}-SAM that was initially coordinated to a porphyrin linker moves away from it. The second simulation has the C_{60}-SAM bound to different Zr-oxo clusters, but with similar starting positions of the fullerene parts. Here, the molecules stay within 3.6 Å of each other and the C_{60}-SAM coordinated to the porphyrin linker stays coordinated to it.

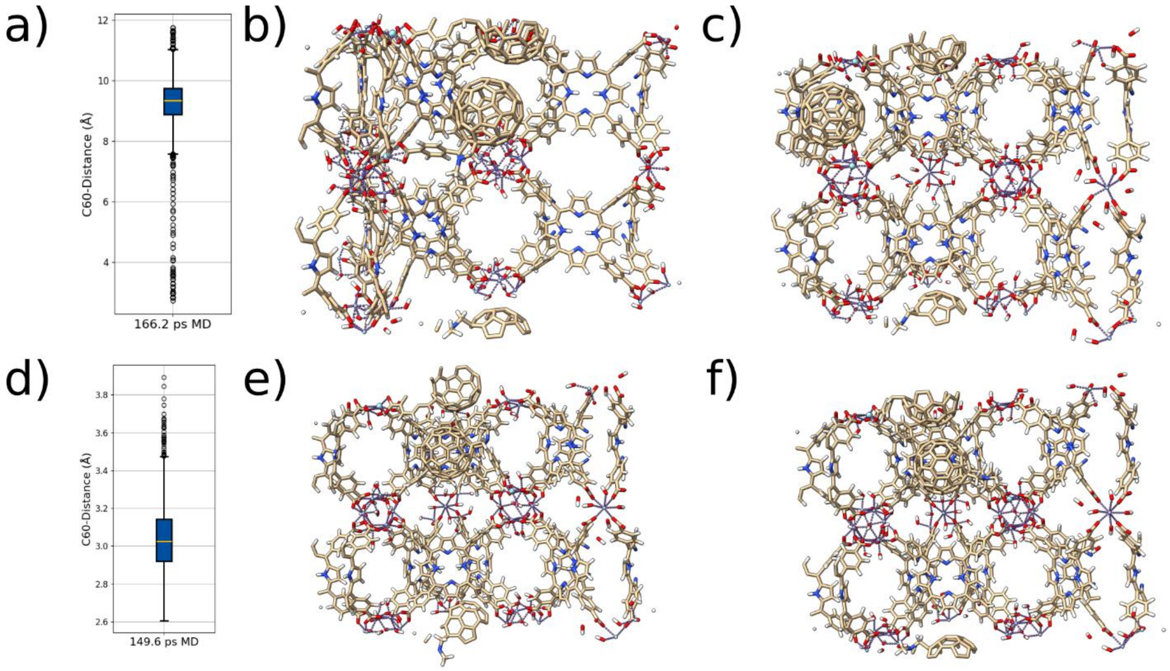

Figure S11. a-c) Boxplot distances between C_{60} moieties alongside initial and final position for a periodic MD simulation of two C_{60}-SAM-COOH molecules bound to Zr-oxo clusters above each other along the porous channel. d-f) Boxplot, and initial and final positions for a periodic MD simulation of two C_{60}-SAM-COOH molecules bound to Zr-oxo clusters oriented diagonally to each other. The Zr atoms that the C_{60}-SAM-COOH molecules are bound to are depicted larger for clarity.

References

(1) Asano, Naomi; Uemura, Sayo; Kinugawa, Tomoya; Akasaka, Hiroaki; Mizutani, Tadashi; Synthesis of Biladienone and Bilatrienone by Coupled Oxidation of Tetraarylporphyrins. *J Org Chem* **2007,** *72*, 5320–5326.
(2) Adler, Alan D.; Longo, Frederick R.; Finarelli, John D.; Goldmacher, Joel; Assour, Jacques; Korsakoff, Leonard; A simplified synthesis for meso-tetraphenylporphine. *J Org Chem* **1967,** *32*, 476–476.
(3) Adler, Alan D.; Longo, Frederick R.; Kampas, Frank; Kim, Jean; On the preparation of metalloporphyrins. *J. Inorg. Nucl. Chem.* **1970,** *32*, 2443–2445.
(4) Jo, Wooseong; Lee, Hyun Seok; Trinh, Tra Phuong; Gupta, Gajendra; Kim, Miyeon; Kim, Ga Young; Kim, Jinho; Kim, Chul Hoon; Lee, Chang Yeon; Sequential Energy and Electron Transfer in Metal–Organic Frameworks. *ACS Appl. Mater. Interfaces* **2024,** *16*, 69479–69491.
(5) Howarth, Ashlee J.; Buru, Cassandra T.; Liu, Yangyang; Ploskonka, Ann M.; Hartlieb, Karel J.; McEntee, Monica; Mahle, John J.; Buchanan, James H.; Durke, Erin M.; Al-Juaid, Salih S., et al.; Postsynthetic Incorporation of a Singlet Oxygen Photosensitizer in a Metal–Organic Framework for Fast and Selective Oxidative Detoxification of Sulfur Mustard. *Chem. Eur. J.* **2017,** *23*, 214–218.
(6) Rouquerol, J.; Llewellyn, P.; Rouquerol, F., Is the bet equation applicable to microporous adsorbents? In *Stud. Surf. Sci. Catal.*, Llewellyn, P. L.; Rodriquez-Reinoso, F.; Rouqerol, J.; Seaton, N., Eds. Elsevier: 2007; Vol. 160, pp 49–56.
(7) Ratkovec, Justin L.; Earley, Justin D.; Kudisch, Max; Kopcha, William P.; Xu, Eve Yuanwei; Knowles, Robert R.; Rumbles, Garry; Reid, Obadiah G.; Electrostatic Work Causes Unexpected Reactivity in Ionic Photoredox Catalysts in Low Dielectric Constant Solvents. *J. Phys. Chem. B* **2025,** *129*, 3895–3901.
